# The prevalence and socio-demographic risk factors of coexistence of stunting, wasting, and underweight among children under five years in Bangladesh: a cross-sectional study

**DOI:** 10.1186/s40795-022-00584-x

**Published:** 2022-08-22

**Authors:** Mohammad Rocky Khan Chowdhury, Md Shafiur Rahman, Baki Billah, Russell Kabir, Nirmala K. P. Perera, Manzur Kader

**Affiliations:** 1 Department of Epidemiology and Preventive Medicine; School of Public Health and Preventive Medicine; Faculty of Medicine, Nursing and Health Sciences, Monash University, Melbourne, VIC Australia; 2 Research Center for Child Mental Development, Hamamatsu University School of Medicine, Shizuoka, Japan; 3 School of Allied Health, Faculty of Health, Education, Medicine and Social Care, Anglia Ruskin University, London, United Kingdom; 4 Nuffield Department of Orthopaedics, Rheumatology, and Musculoskeletal Sciences, University of Oxford, Oxford, United Kingdom; 5 Department of Medicine, Clinical Epidemiology Division, Karolinska Institutet, Maria Aspmans gata 30A, 17164 Solna, Stockholm, Sweden

**Keywords:** BDHS, Child anthropometry, Growth failure, Under 5 children, Binomial regression, Bangladesh

## Abstract

**Background:**

Childhood stunting, wasting and underweight are significant public health challenges. There is a gap in knowledge of the coexistence of stunting, wasting, and underweight among children under five years (under-5) in Bangladesh. This study aims to (i) describe the prevalence of the coexistence of stunting, wasting, and underweight and ii) examine the risk factors for the coexistence of stunting, wasting, and underweight among children under-5 in Bangladesh.

**Methods:**

This study included 6,610 and 7,357 under-5 children from Bangladesh Demographic Health Surveys (BDHS) 2014 and 2017/18, respectively. The associations between the coexistence of stunting, wasting, and underweight and independent variables were assessed using the Chi-square test of independence. The effects of associated independent variables were examined using negative binomial regression.

**Results:**

The prevalence of coexistence of stunting, wasting, and underweight gradually declined from 5.2% in 2014 to 2.7% in 2017/18. Children born with low birth weight ((adjusted incidence rate ratios, aIRR) 2.31, 95% CI 1.64, 3.24)); children of age group 36–47 months (aIRR 2.26, 95% CI 1.67, 3.08); children from socio-economically poorest families (aIRR 2.02, 95% CI 1.36, 2.98); children of mothers with no formal education (aIRR 1.98, 95% CI 1.25, 3.15); and children of underweight mothers (aIRR 1.73, 95% CI 1.44, 2.08) were the most important risk factors. Further, lower incidence among children with the coexistence of stunting, wasting, and underweight was observed in the 2017–18 survey (aIRR 0.59, 95% CI 0.49, 0.70) compared to children in the 2014 survey.

**Conclusions:**

One out of thirty-five under-5 children was identified to have coexistence of stunting, wasting, and underweight in Bangladesh. The burden of coexistence of stunting, wasting, and underweight was disproportionate among children born with low birth weight, socio-economically poorest, a mother with no formal education, and underweight mothers, indicating the need for individual, household, and societal-level interventions to reduce the consequences of coexistence of stunting, wasting, and underweight.

**Supplementary Information:**

The online version contains supplementary material available at 10.1186/s40795-022-00584-x.

## Background

Childhood stunting, wasting, and underweight are significant public health challenges. These three forms together contribute to more than half of global deaths among children under five years (under-5), with the majority in low- and middle-income countries [1]. The coexistence of stunting, wasting, and underweight is prevalent in 124 countries, with 41 severely affected [2]. Bangladesh currently experiences high prevalence of growth failure among its under-5 population, with 40% of children affected by one or more forms of stunting, wasting or underweight and attributing to over 50% of deaths in children under-5 [3, 4]. Critically, more than 30% of children under-5 suffer coexistence of multiple concurrent forms of stunting, wasting, and underweight [4]. Children with the coexistence of such have a 12-fold elevated mortality risk compared to healthy children [5]. Further, the degree of cognitive impairment, impairments to thymic development, decreased growth failure peripheral lymphocyte count, and increased susceptibility to infections are directly related to the severity and co-occurrence of stunting, wasting, or underweight [6].

Bangladesh is one of the world's most densely populated and one of the world's most vulnerable countries due to the adverse effects of climate change and the rise in sea levels. It faces formidable economic challenges, slower progress in poverty reduction, nutritional challenges, especially for women and children, poor access to health, resources, and service; governance issues, and the influx of Rohingya refugees from Myanmar, of whom around 1 million are now in Bangladesh [7]. These have caused detrimental effects on agriculture, consumer price, nutritional status, health coverage, and economic activity [7].

Although child growth failure rates in Bangladesh have declined since the 1990s, progress in tackling all forms of such problems remains unacceptably slow [8]. There are multi-faced risk factors for disaggregated traditional indicators (i.e., stunting, wasting, or underweight). Its ranges from access to nutrients, socio-demographic characteristics, access to healthcare, and geographical location [3, 9–12]. However, assessing risk factors for the combination of three major indicators should be focused on as stunting, wasting, and underweight are all associated with increased mortality, especially when all are present in the same child [6]. The knowledge regarding the coexistence of stunting, wasting, and underweight and its associated factors using large nationally representative samples are yet to be fully uncovered in Bangladesh. It can help to inform context-specific evidence-based prevention strategies. According to some recent evidence, age, sex, and food insecurity have been linked to the coexistence of stunting and wasting [13–15]. In addition, children who are both wasted and stunted are also underweight and have a high risk of death [13]. There is a knowledge gap in evaluating the factors associated with the coexistence of stunting, wasting, and underweight in Bangladesh. However, this study has considered already known aetiology to identify the associated factors of coexistence of stunting, wasting, and underweight among under-5 children in Bangladesh, investigating the change of direction of these factors using more recent data, especially in the context of the coexistence of stunting, wasting and underweight, might help to revise important policy decision-making. Consequently, the present study aims to (i) identify the prevalence of the coexistence of stunting, wasting, and underweight using nationally representative samples of two most recent consecutive surveys (i.e., 2014 and 2017–18), and ii) examine the risk factors for the coexistence of stunting, wasting and underweight among children under-5 in Bangladesh.

## Methods

### Data source

This study pooled the last two most recent consecutive waves of nationally representative cross-sectional data of non-institutional residing Bangladeshi adults and children from the Bangladesh Demographic Health Surveys (BDHS) 2014 and 2017–18. The BDHS collects health and nutritional indicators data using a standard questionnaire with a 99% response rate on average. Details of the survey questionnaire, sample design, and data collection procedure can be found in the BDHS 2014 and 2017–18 reports [16, 17] and the Additional file 1. The data collection of the 2014 survey was done between 28 June 2014 and 9 November 2014, and the 2017–18 survey between 24 October 2017 and 15 March 2018 [16, 17].

**Fig. 1:**
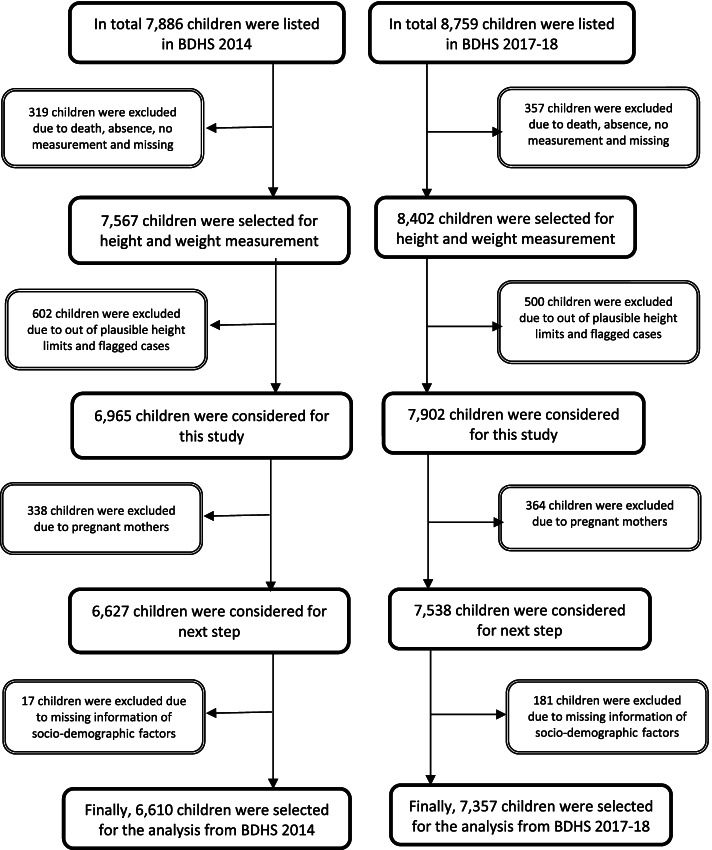
Schematic presentation of sample size selection

The BDHS surveys use two-stage stratified sampling techniques to select primary sampling units (PSUs) and households using probability proportional to their size and an equal probability systematic sampling technique, respectively [16, 17]. The enumeration areas (clusters) were taken from the 2011 censuses compiled by the Bangladesh Bureau of Statistics and were considered the PSUs [16, 17]. This multistage sampling technique, including its sampling weight, helps to reduce potential sampling bias. Information of all ever-married women aged 15–49 years from the pre-selected households was collected without replacement and change in the implementing stage to prevent selection bias. Children born from January 2009 or later and aged under five years at the time of the survey were considered eligible for height and weight measurements. A total of 7,886 (BDHS 2014) and 8,759 (BDHS 2017/18) children met the eligibility criteria, and 6,610 (BDHS 2014) and 7,357 (BDHS 2017/18) children had complete and credible anthropometric and socio-demographic data (Fig. 1).

### Outcome variables and operational definitions

The primary outcome was the coexistence of stunting, wasting, and underweight among under-5 children in Bangladesh. A child was considered to be stunted (short stature for age), wasted (dangerously thin) and underweight (underweight for age) if their height-for-age, weight-for-height, and weight-for-age indices were ≤ -2 standard deviations (SDs) of the World Health Organization (WHO) reference population median [18]. Stunting is not only a cumulative effect specific to undernutrition but social and environmental factors that imply limited physical growth and general development over a long period. Wasting is a form of acute growth failure resulting from poor dietary intake, frequent infections, or diarrhoea. For underweight—a very short infant or child will be of low weight, often underweight. This is a consequence of short stature and small skeletal frame size, which may be due to many non-nutritional factors [18, 19]. Implausible values while estimating child stunting, wasting, and underweight was defined based on the WHO 2006 standards flag limits of unitless z-score: stunting: < -6 or > 6; wasting: < -5 or > 5; and underweight: < -6 or > 5 [19]. The stunting, wasting, and underweight were re-coded dichotomously: 1 = stunted, wasted, or underweighted and 0 = normal/healthy children. After that, the responses of all three indicators were added, resulting in a score ranging from 0 to 3. The scores were again recategorized as 0 for normal, 1 stand for children with a single dimension (either stunting, wasting, or underweight), and 2 for children with co-occurrence of any two indicators (i.e., either stunting and wasting, stunting, and underweight or wasting and underweight) and 3 for children with the coexistence of stunting, wasting and underweight (Additional file 2). Further, overweight children were considered healthy while addressing the outcome of interest and were not excluded from the study.

### Independent variables

A selection of maternal and child, households, and contextual risk factors of interest was identified from relevant literature [3, 4, 8]. Maternal and child characteristics include mother's education (no formal education, primary, secondary, higher); mother's working status (currently not working, presently working); mother's body mass index (underweight, normal, overweight); mother's religion (Islam, others: Hinduism, Buddhism, Christianity); children's age (0–11 months, 12–23 months, 24–35 months, 36–47 months, 48–59 months); sex of child (male, female); birth order (first, second, third, fourth and above); breastfeeding initiation (within 1 h, after 1 h); and birth weight (normal/average, small, not weighted). Household characteristics were the age of the household head (15–34 years, 35–54 years, 55–74 years, and 75 and above); the sex of the household head (male, female); watching television (not at all/do not know, less than once a week, at least once a week); and wealth index (poorest, poorer, middle, richer, richest). The contextual factor was the place of residence (urban, rural).

In low-income countries, babies are often born at home without proper measurement of birth weight. Actual weight at birth was reported for less than 50% of cases [20]. Therefore, all DHS in developing countries retrospectively collect information on the baby's size at birth based on the mother's perception as a proxy of birth weight by asking the question, "was the newborn very large, larger than average, average, smaller than average, or very small?" Approximately 75% of mothers can correctly report their baby's size at birth; therefore, a mother's recall might be considered a valid but weak proxy measure of birth weight [21–23]. The wealth index or socio-economic status was constructed using information about household assets that were collected in BDHSs. The data on household assets included ownership of durable goods (e.g., televisions and bicycles) and dwelling characteristics (e.g., source of drinking water, sanitation facilities, cooking facilities, and construction materials). Principal component analysis was performed to assign individual household wealth scores. These weighted values were then summed and rescaled to range from 0–1, and each household was assigned into quintiles: the first quintile: poorest; the second quintile: poorer; the third quintile: middle class; the fourth quintile: richer, and the fifth quintile: richest [16, 17].

### Statistical analysis

Descriptive statistics were used to describe socio-demographic characteristics. The prevalence of coexistence of stunting, wasting, and underweight and its association with independent variables were assessed using crosstab analysis and Chi-square test. Prevalence estimates considered the complex survey design and sampling weights. In all analyses, the significance level was set at *P* < 0.05 (2-tailed). Adjusted models were developed to analyze the appropriate binary value for the coexistence of stunting, wasting, and underweight among children under-5. Before executing the adjusted model, the BDHS 2014 and the BDHS 2017–18 data sets were appended. This big dataset will help in the credible assessment of associated factors of the coexistence of stunting, wasting, and underweight. All independent variables except those found insignificant in the bivariate analysis (Chi-square test) were simultaneously entered into the negative binomial regression models for adjustment. A negative binomial regression model was used due to unequal dispersion properties, i.e., mean ≠ variance and for the occurrence of rare cases (< 10%). The strength of associations was assessed using incidence rate ratios (IRR). Further, 95% confidence intervals (CIs) were used for significance testing. All statistical analyses were performed using Stata version 14.2 and sample weighting based on the complex design of the BDHSs was considered. Potential clustering was dealt with the Stata command "svyset" that incorporated cluster variable and sampling unit.

## Results

About 15% of mothers had no formal education, 25% of mothers were currently working, and 23% were underweight. About 40% of children came from a family with poor socioeconomic status, and 68% lived in a rural area. About 41% of children were less than 23 months, and 52% were males (Table 1).

**Table 1:** Background characteristics of the children

Factors	Survey year 2014		Survey year 2017/2018	
	**Frequency**	**(%)**	**Frequency**	**(%)**
**Mother's education**				
No education	1,010	15.3	521	7.1
Primary	1,823	27.6	2,098	28.5
Secondary	3,067	46.4	3,498	47.5
Higher	710	10.7	1,240	16.9
**Mother's working status**				
Currently not working	4,937	74.7	4,375	59.5
Currently working	1,673	25.3	2,982	40.5
**Mother's BMI**				
Underweight	1,506	22.8	1,108	15.0
Normal	3,825	57.9	4,339	59.0
Overweight	1,279	19.3	1,910	26.0
**Mother's religion**				
Islam	6,060	91.7	6,712	91.2
Others	550	8.3	645	8.8
**Children's age (in months)**				
0–11	1,335	20.2	1,694	23.1
12–23	1,392	21.1	1,525	20.7
24–35	1,334	20.2	1,404	19.1
36–47	1,280	19.4	1,311	17.8
48–59	1,269	19.2	1,423	19.3
**Sex of child**				
Male	3,413	51.6	3,858	52.4
Female	3,197	48.4	3,499	47.6
**Birth order**				
First	2,525	38.2	2,727	37.1
Second	1,998	30.2	2,431	33.0
Third	1,057	16.0	1,261	17.1
Fourth and above	1,030	15.6	938	12.8
**Size of child at birth **^**a, b**^				
Normal/average	3,812	93.9	1,784	38.6
Small	248	6.1	325	7.0
Not weighted			2,518	54.4
**Age of household head (in years)**				
15–34	2,361	35.7	2,465	33.5
35–54	2,810	42.5	3,101	42.2
55–74	1,233	18.6	1,583	21.5
75 and above	206	3.2	208	2.8
**Sex of household head**				
Male	5,982	90.5	6,461	87.8
Female	628	9.5	896	12.2
**Television watching**				
Not at all/do not know	2,707	40.9	2,783	37.8
Less than once a week	598	9.1	658	9.0
At least once a week	3,305	50.0	3,916	53.2
**Wealth index **^**c**^				
Poorest	1,417	21.4	1,621	22.0
Poorer	1,231	18.6	1,476	20.1
Middle	1,308	19.8	1,325	18.0
Richer	1,366	20.7	1,479	20.1
Richest	1,288	19.5	1,456	19.8
**Place of residence**				
Urban	2,107	31.9	2,520	34.3
Rural	4,503	68.1	4,837	65.7
**Total**	6,610	100.0	7,357	100.0

^a^, *n* = 4,060 in BDHS 2014 and *n* = 4,627 in BDHS 2017/18

^b^, children born with less than 2500 g were considered as small

^c^, an aggregated index based on household assets

### Prevalence of the coexistence of stunting, wasting, and underweight

**Fig. 2:**
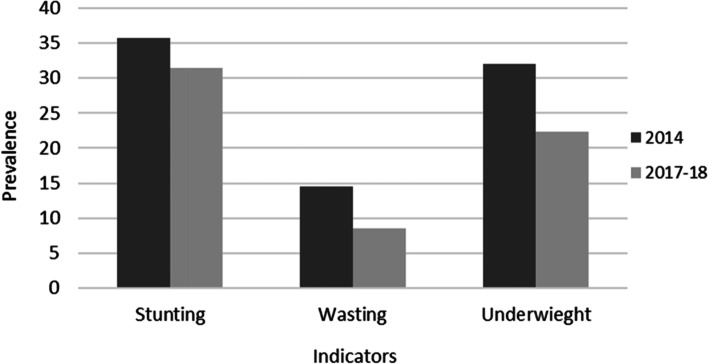
Trends of the prevalence of stunting, wasting and underweight

The prevalence of stunting, wasting, and underweight declined by 4%, 6%, and 10%, respectively from 2014 to 2018 (Fig. 2). For the survey year 2014, the prevalence of coexistence of stunting, wasting, and underweight was 5% which declined to 3% in the survey year 2017/18 (Table 2). In both 2014 and 2017/18 surveys, the prevalence of coexistence of stunting, wasting, and underweight was high in children of underweight mothers (8% vs. 6%), children of mothers with no formal education (8% vs. 5%), children with low birth weight (11% vs. 4%), and from poorest families (8% vs. 4%) (Table 2).

**Table 2:** Prevalence of coexistence of stunting, wasting and underweight among children under-5

Factors	Survey year 2014				Survey year 2017/18			
	**Number**	**Prevalence (95% CI)**	***P* values**	**Cramér's V**	**Number**	**Prevalence (95% CI)**	***P* values**	**Cramér's V**
**Mother's education**								
No education	84	7.8 (5.9, 10.2)	0.0007	0.069	28	5.0 (3.3, 7.4)	< 0.001	0.070
Primary	120	5.6 (4.4, 7.0)			73	3.6 (2.8, 4.6)		
Secondary	143	4.5 (3.7, 5.5)			96	2.6 (2.1, 3.2)		
Higher	21	3.1 (1.9, 5.1)			9	0.7 (0.4, 1.5)		
**Mother's working status**								
Currently not working	246	4.6 (3.9, 5.4)	0.001	0.043	106	2.3 (1.9, 2.9)	0.012	0.027
Currently working	122	7.0 (5.5, 8.9)			100	3.3 (2.7, 4.1)		
**Mother's BMI**								
Underweight	144	8.4 (6.8, 10.4)	< 0.001	0.102	62	5.8 (4.5, 7.6)	< 0.001	0.075
Normal	190	5.0 (4.1, 6.0)			112	2.5 (2.0, 3.0)		
Overweight	34	2.2 (1.5, 3.2)			32	1.7 (1.1, 2.5)		
**Mother's religion**								
Islam	343	5.3 (4.6,6.1)	0.466	-0.013	187	2.8 (2.4,3.2)	0.749	0.001
Others	25	4.3 (2.6,7.2)			19	2.6 (1.6,4.0)		
**Children's age (in months)**								
0–11	40	2.8 (2.0, 4.1)	0.003	0.060	42	3.0 (2.2, 4.1)	0.009	0.047
12–23	91	6.1 (4.6, 8.1)			47	3.1 (2.3, 4.3)		
24–35	72	5.1 (3.9, 6.7)			47	3.5 (2.6, 4.8)		
36–47	87	6.2 (4.9, 7.9)			46	2.9 (2.2, 4.0)		
48–59	78	5.8 (4.4, 7.6)			24	1.4 (0.9, 2.2)		
**Sex of child**								
Male	206	5.3 (4.4, 6.4)	0.748	-0.021	117	3.0 (2.5, 3.6)	0.199	-0.015
Female	162	5.1 (4.2, 6.1)			89	2.5 (1.9, 3.1)		
**Birth order**								
First	123	4.4 (3.5, 5.6)	0.018	0.050	38	3.6 (2.5, 5.2)	0.313	0.030
Second	96	4.8 (3.7, 6.2)			31	2.5 (1.7, 3.6)		
Third	67	5.7 (4.2, 7.5)			61	2.4 (1.9, 3.1)		
Fourth and above	82	7.5 (5.7, 9.7)			76	2.8 (2.2, 3.7)		
**Size of child at birth **^**a**^								
Normal/average	174	4.3 (3.5, 5.2)	0.0001	0.078	26	1.5 (1.0, 2.3)	0.002	0.048
Small	29	10.8 (6.9, 16.5)			14	4.1 (2.3, 7.4)		
Not weighted					77	3.1 (2.4, 3.9)		
**Age of household head (in years)**								
15–34	120	5.2 (4.2,6.4)	0.202	0.041	77	3.1 (2.4,4.0)	0.157	0.026
35–54	182	5.6 (4.7,6.8)			80	2.6 (2.0,3.2)		
55–74	61	4.9 (3.5,6.9)			39	2.3 (1.6,3.2)		
75 and above	5	1.6 (0.5,5.2)			10	4.9 (2.4,9.9)		
**Sex of household head**								
Male	342	5.3 (4.6,6.1)	0.413	-0.020	190	2.9 (2.5,3.4)	0.009	-0.023
Female	26	4.4 (3.0,6.6)				1.4 (0.8,2.4)		
**Television watching**								
Not at all/do not know	191	6.6 (5.4, 8.0)	0.001	0.056	88	3.0 (2.4, 3.7)	0.006	0.039
Less than once a week	34	4.8 (3.2, 7.2)			29	4.5 (3.0, 6.5)		
At least once a week	143	4.1 (3.4, 5.0)			89	2.3 (1.8, 2.9)		
**Wealth index b**								
Poorest	132	8.3 (6.8, 10.2)	< 0.001	0.101	61	4.0 (3.1,5.1)	0.0003	0.058
Poorer	82	6.2 (4.7, 8.1)			53	3.2 (2.4, 4.3)		
Middle	62	3.8 (2.8, 5.3)			42	2.9 (2.1, 4.0)		
Richer	58	4.3 (3.2, 5.8)			32	2.2 (1.5, 3.3)		
Richest	34	2.9 (2.0, 4.2)			18	1.1 (0.7, 1.9)		
**Place of residence**								
Urban	95	4.5 (3.5, 5.8)	0.238	0.031	62	2.4 (1.9, 3.1)	0.293	0.014
Rural	273	5.4 (4.6, 6.5)			144	2.9 (2.4, 3.4)		
**Total**	368	5.2 (4.5, 6.0)			206	2.7 (2.4, 3.2)		

^a^, children born with less than 2500 g were considered as small

^b^, an aggregated index based on household assets

### Risk factors

Results from regression analysis showed that the most influential risk factors for the coexistence of stunting, wasting, and underweight were children born with low birth weight (adjusted IRR (aIRR) 2.31, 95% CI 1.64, 3.24, *p* = 0.010); children of age group 36–47 months (aIRR 2.26, 95% CI 1.67, 3.08, *p* < 0.001); children from socio-economically poorest families (aIRR 2.02, 95% CI 1.36, 2.98, *p* < 0.001); children of mothers with no formal education (aIRR 1.98, 95% CI 1.25, 3.15, *p* = 0.004); and children of underweight mothers (aIRR 1.73, 95% CI 1.44, 2.08, *p* < 0.001). Further, lower incidence among children with the coexistence of stunting, wasting, and underweight was observed in the 2017–18 survey (aIRR 0.59, 95% CI 0.49, 0.70, *p* < 0.001) compared to children in the 2014 survey (Table 3).

**Table 3:** Risk factors of the coexistence of stunting, wasting and underweight

Factors	Unadjusted IRR (95% CI)	*P* values	Adjusted IRR (95% CI)	*P* values
**Mother’s education **^**a, b**^				
No education	4.75 (3.18–7.11)	< 0.001	1.98 (1.25–3.15)	0.004
Primary	3.20 (2.18–4.70)	< 0.001	1.62 (1.06–2.48)	0.026
Secondary	2.37 (1.62–3.46)	< 0.001	1.59 (1.06–2.38)	0.024
Higher	1.00		1.00	
**Mother's working status **^**a, b**^				
Currently not working	1.00		1.00	
Currently working	1.26 (1.06–1.49)	0.007	1.27 (1.06–1.52)	0.008
**Mother's BMI **^**a, b**^				
Underweight	2.13 (1.78–2.54)	< 0.001	0.61 (0.46–0.82)	0.001
Normal	1.00		1.00	
Overweight	0.56 (0.43–0.73)	0.001	0.79 (0.53–1.20)	0.274
**Children's age (in months) **^**a, b**^				
0–11	1.00		1.00	
12–23	2.21 (1.64–2.97)	< 0.001	2.11 (1.56–2.85)	< 0.001
24–35	2.05 (1.52–2.77)	< 0.001	2.07 (1.52–2.82)	< 0.001
36–47	2.43 (1.81–3.28)	< 0.001	2.26 (1.67–3.08)	< 0.001
48–59	2.09 (1.55–2.84)	< 0.001	2.06 (1.51–2.81)	< 0.001
**Sex of child **^**a, b**^				
Male	1.00		1.00	
Female	0.84 (0.71–0.99)	0.042	0.90 (0.77–1.07)	0.242
**Birth order **^**a, b**^				
First	1.00		1.00	
Second	0.93 (0.76–1.15)	0.536	0.97 (0.78–1.20)	0.756
Third	1.11 (0.87–1.42)	0.382	0.98 (0.76–1.27)	0.879
Fourth and above	1.61 (1.28–2.02)	< 0.001	1.17 (0.90–1.52)	0.248
**Size of child at birth **^**b**^				
Normal/average	1.00		1.00	
Small	2.11 (1.51, 2.93)	< 0.001	2.31 (1.64, 3.24)	0.010
**Sex of household head **^**a, b**^				
Male	1.00		1.00	
Female	0.64 (0.47–0.88)	0.006	0.72 (0.53–1.00)	0.049
**Television watching **^**a, b**^				
Not at all/do not know	1.00		1.00	
Less than once a week	0.99 (0.75–1.30)	0.927	1.17 (0.88–1.56)	0.278
At least once a week	0.63 (0.53–0.75)	< 0.001	1.09 (0.87–1.36)	0.450
**Wealth index **^**a, b**^				
Poorest	3.35 (2.47–4.55)	< 0.001	2.02 (1.36–2.98)	< 0.001
Poorer	2.63 (1.91–3.62)	< 0.001	1.72 (1.17–2.53)	0.005
Middle	2.08 (1.49–2.90)	< 0.001	1.34 (0.93–1.94)	0.119
Richer	1.66 (1.18–2.34)	0.003	1.33 (0.93–1.88)	0.115
Richest	1.00		1.00	
**Place of residence **^**a, b**^				
Urban	1.00		1.00	
Rural	1.31 (1.09–1.58)	0.004	0.82 (0.66–1.03)	0.092
**Survey year **^**a, b**^				
2014	1.00		1.00	
2017–18	0.51 (0.43–0.60)	< 0.001	0.59 (0.49–0.70)	< 0.001

^a^, adjusting all significant variables including child sex and place of residence in the regression analysis except size of child at birth

^b^, simultaneously adjusting all significant variables including child sex and place of residence in the regression analysis

Three separate models, such as regression Model I, Model II, and Model III, were carried out to highlight the significant variables that controlled for maternal characteristics, child characteristics, and household and contextual characteristics, respectively (Additional file 2). Further, for both surveys, the adjustments for regression models were made, taking into account all significant variables (Additional file 2).

## Discussion

The current study highlights that the prevalence of stunting, wasting, and underweight declined by 4%, 6%, and 10%, respectively from 2014 to 2018. Between the earlier and later surveys, the prevalence of stunting declined less than the prevalence of wasting and underweight. The finding suggests that stunted children (in the first two years) may be chronically have a disadvantage to regain height later in childhood while wasting and underweight are acute cases often related to the inadequate quantity and quality of food [24]. Food insecurity and other insecurities cause emotional and physiological stress, and this can cause stunting [25]. However, the causes of undernutrition are multidimensional such as, immediate causes (inadequate dietary intake, acute disease), underlining causes (household food insecurity, unhealthy environment, inadequate healthcare service, and feeding practice), and basic causes (education, employment, income, technology, cultural, economic and political context) [26, 27]. This may create many challenges and take a long time in understanding the condition and finding solutions through interventions and policies. On the other hand, the prevalence of a minimum acceptable diet (MAD) increases from 23% in 2014 to 35% in 2018 in Bangladesh, which helped improve the condition of wasting and being underweight over time [16, 17].

One of the key findings of this study is approximately 3% of children under-5 experience coexistence of stunting, wasting, and underweight which can have a detrimental impact on their short- and long-term health. India reports a very high figure, with approximately one in ten children under-5 reporting coexistence of stunting, wasting, and underweight [28]. Compared to other poor-income countries like Malawi (2%) and Ethiopia (4%) [29, 30], the prevalence of coexistence of stunting, wasting, and underweight is high in Bangladesh. Limited resources at the National Nutrition Services (NNS) in Bangladesh may result in limited coverage and quality of interventions. Frequent changes in leadership, coordination, capacity, and workload-related challenges the NNS face have hampered the implementation of nutrition interventions [31]. However, we observed the coexistence of stunting, wasting, and underweight among children declined 2% in 2017/18 from 5% in 2014, indicating that current interventions might be effective. Therefore, leadership, stability, and resources at the NNS can provide further coverage of high-quality interventions further to decrease the coexistence of stunting, wasting, and underweight in children under-5.

This study found that the relative risk of coexistence of stunting, wasting, and underweight increased by 130% in children with low birth weight compared to normal weight. Children with low birth weight experience growth failure during early childhood, increasing the risk of long-term complications like diarrheal and lower respiratory infections, sleep apnea, jaundice, anemia, chronic lung disorders, fatigue, and loss of appetite [20]. Low birth weight was a risk factor for the coexistence of stunting, wasting, and underweight, and our results concur with Ramakrishnan (2004) [32]. Children of the older age group (36–47 months) had a 2.5 times higher risk of coexistence of stunting, wasting, and underweight than the youngest children (less than 1 year). Das and Gulshan (2017) found older children had a high risk of stunting ((odds ratio (OR): 1.5)) and a lower risk of wasting in Bangladesh [33]. In that case, the estimated risk of the coexistence of stunting, wasting, and underweight among older children was higher compared to previous study findings. After the second year of life, children in Bangladesh tend to have the same diet as the family and breast milk. However, they are often allowed to eat the food themselves, and they do not always have access to adequate amounts of solid food, which might contribute to several anthropometric failure, such as, stunting, wasting or underweight [34]. Poorer socioeconomic status [3] is another risk factor that contributes coexistence of stunting, wasting, and underweight, and our findings concur, demonstrating the complex nature of this public health issue.

The risk of having coexistence of stunting, wasting, and underweight increased by 98% in children of mothers with no formal education. Lack of maternal education was assessed as an influential risk factor for child stunting, wasting, or underweight in previous studies in Bangladesh and other developing countries [8, 35–37]. Current evidence also showed 5% of children of mothers with no formal education were suffering from the coexistence of stunting, wasting, and underweight. The parallel state of poor maternal educational and socio-economic status in households might affect children with critical nutritional hazards due to knowledge gaps and the inability to provide an appropriate diet [38]. Also, the coexistence of stunting, wasting, and underweight among children increased by 95% for those born to underweight mothers. Likely because mothers are malnourished due to the emotional and physiological impact of food insecurity, poverty, and micronutrient deficiencies [39]. Investing in the maternal and child healthcare system, and increasing the participation of underprivileged people in income-generating activities can improve the nutritional status of children as well as other physical development. Further, improving women's education can increase family income and access to a better quality of diet, consequently improving children's health [40]. Increasing education opportunities for females, especially in rural areas, is recommended [8].

The study findings also showed that a higher incidence of coexistence of stunting, wasting, and underweight was observed in children in the 2014 BDHS survey (children born between 2009 and 2014) than those in the 2017–18 survey (children born between 2014 and 2017). Nutritional changes include a rise in household assets, improvements in parental education, food security, and increasing dietary diversity. It also consists of reducing open defecation, improvements in prenatal and birth delivery care, family reproductive factors (birth order and birth intervals), maternal height and weight, and increasing agricultural production. GO-NGO-led nutritional programs might significantly reduce the incidence of coexistence of stunting, wasting, and underweight [41]. However, the country still faces significant challenges in providing equitable access to health, nutrition, and population services.

This study also suggests some policy implications and interventions to prevent and treat the coexistence of stunting, wasting, and underweight. Routine national and subnational level nutrition surveys such as demographic health surveys (DHS) and Multiple Indicator Cluster Surveys (MICSs) need to be modified to include the coexistence of stunting, wasting, and underweight to inform the program policy decision-making. Routine monitoring of the prevalence of coexistence of stunting, wasting, and underweight would be required to inform effective detection and treatment [42]. Community engagement and coexistence of stunting, wasting, and underweight screening could also be expanded in innovative methods by enrolling additional expertise and resources [43]. Innovative and early markers should be developed to predict, identify, and monitor children at short-term and long-term consequences due to the coexistence of stunting, wasting, and underweight [44]. Maternal factors from adolescence through pregnancy need to be searched that adversely affect utero and postnatal child who is living with stunting, wasting, and underweight [44]. Therapeutic interventions (e.g., ready-to-use therapeutic foods) must be reviewed and adjusted to ensure that the children at the highest mortality risk due to the coexistence of stunting, wasting, and underweight are included. Comprehensive nutrition programmes must be developed to pursue Sustainable Development Goal (SDG) 2.2, to end stunting, wasting, and underweight by 2030 [41].

The use of multiple nationally representative household survey data points with a high response rate was the strength of this study. The survey questions were validated and established. Although suitable statistical tools like Negative Binomial Regression were used to assess the risk factors, the cross-sectional nature of the data was not sufficient to establish a causal relationship between risk factors and the dependent variables. Further, data on potential confounders like diet, food insecurity, and parental smoking behavior were unavailable Child’s birth size from mothers’ recall was used as a proxy of actual measurement of size at birth due to unavailability of measure data in BDHS, and thus should be used with caution. The BDHS data were collected retrospectively and self-reported; underreporting, information bias, and recall bias might be possible.

## Conclusion

One out of thirty-five Bangladeshi children under-5 were identified to have coexistence of stunting, wasting, and underweight in Bangladesh. Risk factors for the coexistence of stunting, wasting, and underweight were multi-faced. Low birth weight, children of older age group (36–47 months), poorest socioeconomic status, lack of maternal education, and children of underweight mothers increase the risk of getting the coexistence of stunting, wasting, and underweight. Although these factors are already known in the etiology of stunting, wasting, and underweight, it needs consistent revision that will help in understanding the trends and magnitude of risk of these factors over time and these factors should be the focus of evidence-based interventions. Our study will provide helpful guidelines for intervention development from the household level to the societal level to reduce short- and long-term health consequences of the coexistence of stunting, wasting, and underweight. Effective and systematic coordination of interventions requires different nutritional programs and policies to support such strategies.

## Supplementary Information

**Additional file 1. **Sample size selection.

**Additional file 2: Table 1.** Estimating scores for a child with different nutritional status. **Table 2**. Variable definitions. **Table 3. **Determinants of coexistence of stunting, wasting and underweight in context of maternal and child characteristics, and household and contextual characteristics.**Table 4.** Risk factors of coexistence of stunting, wasting and underweight among children under-5 (in separate assessment from 2014 and 2017-18 surveys).

## Data Availability

The data underlying the results presented in the study are publicly accessible and available from the DHS website (https://dhsprogram.com/data/available-datasets.cfm). The name of the dataset is BangladeshDemographic and Health Survey (BDHS).

## Abbreviations

BDHS: Bangladesh Demographic Health Survey
BMI: Body Mass Index
CI: Confidence Interval
DHS: Demographic Health Survey
IRR: Incidence Rate Ratio
MAD: Minimum Acceptable Diet
MICS: Multiple Indicator Cluster Surveys
NNS: National Nutrition Services
PSU: Primary Sampling Units
SDG: Sustainable Development Goal
WHO: World Health Organization
