# Supplementary material for: The prevalence and socio-demographic risk factors of coexistence of stunting, wasting, and underweight among children under five years in Bangladesh: a cross-sectional study
Source: BMC Nutr. 2022 Aug 22;8:84. doi: 10.1186/s40795-022-00584-x (PMC9394024; doi:10.1186/s40795-022-00584-x)
Supplement: Supplementary file 1 — Additional file 1. Sample size selection. [file 40795_2022_584_MOESM1_ESM.docx]

**Sample size selection**

Bangladesh Demographic and Health Survey (BDHS) is a nationwide survey. It is a nationally representative sample with approximately 20,250 selected households in the 2017/18 survey and 18,000 households in the 2014 survey. All ever-married women aged 15-49 who of the selected households who spent the night in the selected households prior to the survey were eligible for individual interviews.

The sampling frame contains information about enumeration areas’ (EAs) location, type of residence (urban or rural), and the selected number of residential households. Administratively, Bangladesh has been divided into eight divisions in the 2017/18 survey and seven divisions in the 2014 survey. Each division was further classified as urban and rural areas. Urban areas were classified into two groups: city corporations and areas other than city corporations. In Bangladesh, 23.3% of households are located in urban areas with 8% being in city corporations, and 15.3% are in areas other than city corporations based on the 2011 census. In a village, an EA usually consists of a village, a group of small villages, or part of a large village. Consequently, each division was stratified into urban city corporations, urban areas other than city corporations, and rural areas, yielding a total of 22 sampling strata in the 2017/18 survey and 20 sampling strata in the 2014 survey. The sample was stratified and selected in two-stage. In the first stage, 675 EAs (227 in urban areas and 448 in rural areas) were independently selected in the 2017/18 survey and 600 EAs (207 in urban areas and 393 in rural) in the 2014 survey using probability proportional to EA size. A list of households was drawn from the selected sample of EAs. For the selected EAs which have more than 200 households, considered large EA and was segmented using probability proportional to the segment size. So, a BDHS cluster is either an EA or a segment of an EA. In the second stage, a fixed number of 30 households from the listing households per cluster were selected with an equal probability systematic selection in both surveys. Interviews were carried out only in the pre-selected households. To prevent bias, no replacements and changes of the pre-selected households were made in the implementing stages. The sample was expected to result in about 20,108 completed interviews with ever-married women age 15-49, 6,763 in urban areas and 13,345 in rural areas in 2017/18 survey, and 17,886 completed interviews, 6,150 in urban areas and 11,736 in rural areas in 2014 survey. Children’s data were drawn by interviewing ever-married women in each survey where under-5 children were available at households.
