# Supplementary material for: The prevalence and socio-demographic risk factors of coexistence of stunting, wasting, and underweight among children under five years in Bangladesh: a cross-sectional study
Source: BMC Nutr. 2022 Aug 22;8:84. doi: 10.1186/s40795-022-00584-x (PMC9394024; doi:10.1186/s40795-022-00584-x)
Supplement: Supplementary file 2 — Additional file 2: Table 1. Estimating scores for a child with different nutritional status. Table 2. Variable definitions. Table 3. Determinants of coexistence of stunting, wasting and underweight in context of maternal and child characteristics, and household and contextual characteristics.Table 4. Risk factors of coexistence of stunting, wasting and underweight among children under-5 (in separate assessment from 2014 and 2017-18 surveys). [file 40795_2022_584_MOESM2_ESM.docx]

**Table 1** Estimating scores for a child with different nutritional status

| **Nutritional indicators** | **Code** | **Stunting** | **wasting** | **underweight** | **Total score** |
| --- | --- | --- | --- | --- | --- |
|  |  | **yes (1)/no (0)** | **yes (1)/no (0)** | **yes (1)/no (0)** |  |
| **Stunning** |  |  |  |  |  |
| No | 0 |  |  |  |  |
| Yes | 1 |  |  |  |  |
| **Wasting** |  |  |  |  |  |
| No | 0 |  |  |  |  |
| Yes | 1 |  |  |  |  |
| **Underweight** |  |  |  |  |  |
| No | 0 |  |  |  |  |
| Yes | 1 |  |  |  |  |
| **Healthy children/no undernutrition** |  | 0 | 0 | 0 | 0 |
| **Only stunting** |  | 1 | 0 | 0 | 1 |
| **Only wasting** |  | 0 | 1 | 0 | 1 |
| **Only underweight** |  | 0 | 0 | 1 | 1 |
| **Stunting & wasting** |  | 1 | 0 | 1 | 2 |
| **Stunting & underweight** |  | 1 | 0 | 1 | 2 |
| **Wasting and underweight** |  | 0 | 1 | 1 | 2 |
| **Stunting, wasting and underweight** |  | 1 | 1 | 1 | 3 |

**Table 2** Variable definitions

| **Variable** | **Type of variable** | **Description** | **Measurement** | **Scale of measurement** | **Number of responses/Missing** |
| --- | --- | --- | --- | --- | --- |
| **Coexistence of stunting, wasting and underweight** | **Dependent variable** | A child was considered to be coexistence of stunting; wasting and underweight if wasting, stunting and underweight were present in the same child. | No, yes | Binary | 14,076 /2,569 |
| **Mother’s education** | Independent variable | Mother educational status | No educated, primary, secondary and higher | Categorical | 14,118 /2,527 |
| **Mother currently working** | Independent variable | Mothers engaged in economic activity at the time of data collection | No, yes | Binary | 13,967/2,678 |
| **Underweight mother** | Independent variable | Mother with <18.5 kg/m^2^ of body mass index | No, yes | Binary | 14,118 /2,527 |
| **Mother’s religion** | Independent variable | Religious belief of mothers. Such as, Islam, Hindu, Christian and Buddhist | Islam, others | Binary | 14,118 /2,527 |
| **Children’s age (in months)** | Independent variable | Age of the children at the time of data collection | 0-11 months, 12-23 months, 24-35 months, 36-47 months, 48-59 months | Categorical | 14,118 /2,527 |
| **Sex of child** | Independent variable | Sex differential of children | Male, female | Binary | 14,118 /2,527 |
| **Birth order** | Independent variable | Birth order is the chronological order of sibling births in a family | One, two, three, four and above | Categorical | 14,118 /2,527 |
| **Low birth weight** | Independent variable | Children were <2.5 kg of weight during birth. The health card was filled in at the time of the birth, if the baby was weighted, or by mother’s recall. Approximately 75% mothers can correctly report their baby's size at birth; therefore mother’s recall is a valid proxy measure of birth weight. | No, yes, not weighted | Categorical | 11,290/5,355 |
| **Age of household head** | Independent variable | Person lead the family age 15 years and above at the time of data collection. | 15-34 years, 35-54 years, 55-74 years, 75 years and above | Categorical | 14,118 /2,527 |
| **Sex of household head** |  | Sex of a person lead the family | Male, female | Binary |  |
| **Television watching** | Independent variable | Mother exposed to mass media through television has been defined as watching television. | Not at all/do not know, less than once a week, at least once a week | Categorical | 14,118 /2,527 |
| **Wealth index** | Independent variable | Wealth index in the DHS surveys is calculated, by the DHS authority, based on information on household characteristics and assets using principal component analysis. Principal component analysis was performed to assign individual household wealth scores. These weighted values were then summed and rescaled to range from 0-1, and each household was assigned into quintiles: the first quintile: poorest (lowest values of the index); the second quintile: poorer; the third quintile: middle class; the fourth quintile: richer and the fifth quintile: richest. | Poorest, poorer, middle, richer, richest | Categorical | 14,118 /2,527 |
| **Place of residence** | Independent variable | Respondent’s place of residence | Urban, rural | Binary | 14,118 /2,527 |

**Table 3** Determinants of coexistence of stunting, wasting and underweight in context of maternal and child characteristics, and household and contextual characteristics

| **Factors** | **Model I** | | **Model II** | | **Model III** | |
| --- | --- | --- | --- | --- | --- | --- |
|  | **Unadjusted IRR (95% CI)** | **P values** | **Adjusted IRR (95% CI)** | **P values** | **Adjusted IRR (95% CI)** | **P values** |
| **Mother’s education** |  |  |  |  |  |  |
| No education | 3.62 (2.41, 5.44) | <0.001 |  |  |  |  |
| Primary | 2.58 (1.75, 3.80) | <0.001 |  |  |  |  |
| Secondary | 2.11 (1.44, 3.08) | <0.001 |  |  |  |  |
| Higher | 1.00 |  |  |  |  |  |
| **Mother’s working status** |  |  |  |  |  |  |
| Currently not working | 1.00 |  |  |  |  |  |
| Currently working | 1.21 (1.02, 1.43) | 0.021 |  |  |  |  |
| **Mother’s BMI** |  |  |  |  |  |  |
| Underweight | 1.98 (1.65, 2.36) | <0.001 |  |  |  |  |
| Normal | 1.00 |  |  |  |  |  |
| Overweight | 0.63 (0.48, 0.82) | 0.001 |  |  |  |  |
| **Children’s age (in month)** |  |  |  |  |  |  |
| 0-11 |  |  | 1.00 |  |  |  |
| 12-23 |  |  | 2.20 (1.63, 2.96) | <0.001 |  |  |
| 24-35 |  |  | 2.02 (1.49, 2.73) | <0.001 |  |  |
| 36-47 |  |  | 2.40 (1.78, 3.24) | <0.001 |  |  |
| 48-59 |  |  | 2.04 (1.50, 2.76) | <0.001 |  |  |
| **Sex of child** |  |  |  |  |  |  |
| Male |  |  | 1.00 |  |  |  |
| Female |  |  | 0.84 (0.71, 0.99) | 0.040 |  |  |
| **Birth order** |  |  |  |  |  |  |
| First |  |  | 1.00 |  |  |  |
| Second |  |  | 0.93 (0.75, 1.15) | 0.515 |  |  |
| Third |  |  | 1.10 (0.86, 1.41) | 0.423 |  |  |
| Fourth and above |  |  | 1.57 (1.25, 1.98) | <0.001 |  |  |
| **Size of child at birth** |  |  |  |  |  |  |
| Normal/average |  |  | 1.00 |  |  |  |
| Small |  |  | 2.12 (1.52, 2.95) | <0.001 |  |  |
| **Sex of household head** |  |  |  |  |  |  |
| Male |  |  |  |  | 1.00 |  |
| Female |  |  |  |  | 0.72 (0.53, 0.99) | 0.042 |
| **Television watching** |  |  |  |  |  |  |
| Not at all/do not know |  |  |  |  | 1.00 |  |
| Less than once a week |  |  |  |  | 1.20 (0.90, 1.58) | 0.206 |
| At least once a week |  |  |  |  | 1.02 (0.82, 1.26) | 0.870 |
| **Wealth index** |  |  |  |  |  |  |
| Poorest |  |  |  |  | 3.56 (2.49, 5.08) | <0.001 |
| Poorer |  |  |  |  | 2.80 (1.96, 4.00) | <0.001 |
| Middle |  |  |  |  | 2.13 (1.50, 3.02) | <0.001 |
| Richer |  |  |  |  | 1.70 (1.20, 2.40) | 0.003 |
| Richest |  |  |  |  | 1.00 |  |
| **Place of residence** |  |  |  |  |  |  |
| Urban |  |  |  |  | 1.00 |  |
| Rural |  |  |  |  | 0.89 (0.63, 1.09) | 0.273 |
| **Survey year** |  |  |  |  |  |  |
| 2014 |  |  |  |  | 1.00 |  |
| 2017-18 |  |  |  |  | 0.50 (0.42, 0.60) | <0.001 |

**Model I** is adjusted for maternal characteristics only

**Model II** is adjusted for child characteristics only

**Model III** is adjusted for household and contextual factors only

**Table 4** Risk factors of coexistence of stunting, wasting and underweight among children under-5 (in separate assessment from 2014 and 2017-18 surveys)

| **Factors** | **Survey year 2014** | | **Survey year 2017/18** | |
| --- | --- | --- | --- | --- |
|  | **Adjusted IRR (95% CI)** | **P values** | **Adjusted IRR (95% CI)** | **P values** |
| **Mother’s education ^A, B^** |  |  |  |  |
| No education | 1.14 (0.66-1.98) | 0.633 | 4.74 (2.05-11.01) | <0.001 |
| Primary | 0.96 (0.57-1.62) | 0.881 | 3.58 (1.68-7.64) | 0.001 |
| Secondary | 1.07 (0.66-1.74) | 0.780 | 2.93 (1.42-6.05) | 0.004 |
| Higher | 1.00 |  | 1.00 |  |
| **Mother’s working status ^A, B^** |  |  |  |  |
| Currently not working | 1.00 |  | 1.00 |  |
| Currently working | 1.36 (1.09-1.69) | 0.007 | 1.13 (0.85-1.51) | 0.393 |
| **Mother’s BMI ^A, B^** |  |  |  |  |
| Underweight | 1.58 (1.26-1.99) | <0.001 | 2.09 (1.52-2.87) | <0.001 |
| Normal | 1.00 |  | 1.00 |  |
| Overweight | 0.49 (0.33-0.74) | 0.001 | 0.79 (0.53-1.20) | 0.274 |
| **Children’s age (in month) ^A, B^** |  |  |  |  |
| 0-11 | 1.00 |  | 1.00 |  |
| 12-23 | 2.10 (1.45-3.06) | <0.001 | 2.07 (1.25-3.44) | 0.005 |
| 24-35 | 1.77 (1.19-2.62) | 0.004 | 2.54 (1.54-4.18) | <0.001 |
| 36-47 | 2.22 (1.51-3.25) | <0.001 | 2.22 (1.32-3.72) | 0.002 |
| 48-59 | 1.99 (1.35-2.93) | 0.001 | 2.13 (1.27-3.58) | 0.004 |
| **Sex of child ^A, B^** |  |  |  |  |
| Male | 1.00 |  | 1.00 |  |
| Female | 0.97 (0.79-1.19) | 0.766 | 0.83 (0.62-1.10) | 0.187 |
| **Birth order ^A, B^** |  |  |  |  |
| First | 1.00 |  | 1.00 |  |
| Second | 1.08 (0.82-1.42) | 0.577 | 0.82 (0.58-1.15) | 0.251 |
| Third | 1.17 (0.85-1.61) | 0.338 | 0.74 (0.48-1.14) | 0.167 |
| Fourth and above | 1.37 (0.99-1.89) | 0.059 | 0.90 (0.58-1.41) | 0.656 |
| **Size of child at birth ^B, C^** |  |  |  |  |
| Normal/average | 1.00 |  | 1.00 |  |
| Small | 2.32 (1.56, 3.46) | <0.001 | 2.38 (1.23, 4.60) | 0.010 |
| **Sex of household head ^A, B^** |  |  |  |  |
| Male | 1.00 |  | 1.00 |  |
| Female | 0.87 (0.58-1.29) | 0.485 | 0.53 (0.30-0.92) | 0.024 |
| **Television watching ^A, B^** |  |  |  |  |
| Not at all/do not know | 1.00 |  | 1.00 |  |
| Less than once a week | 0.90 (0.60-1.33) | 0.584 | 1.71 (1.12-2.63) | 0.013 |
| At least once a week | 1.05 (0.78-1.40) | 0.762 | 1.15 (0.81-1.64) | 0.423 |
| **Wealth index ^A, B, D^** |  |  |  |  |
| Poorest | 2.10 (1.29-3.41) | 0.003 | 1.93 (0.98-3.80) | 0.057 |
| Poorer | 1.73 (1.07-2.80) | 0.024 | 1.73 (0.90-3.34) | 0.103 |
| Middle | 1.14 (0.72-1.81) | 0.582 | 1.75 (0.93-3.29) | 0.084 |
| Richer | 1.28 (0.84-1.97) | 0.252 | 1.46 (0.78-2.70) | 0.235 |
| Richest | 1.00 |  | 1.00 |  |
| **Place of residence ^A, B^** |  |  |  |  |
| Urban | 1.00 |  | 1.00 |  |
| Rural | 0.81 (0.61-1.07) | 0.137 | 0.89 (0.62-1.30) | 0.554 |

A, adjusting all significant variables including child age, sex and place of residence in the regression analysis except size of child at birth

B, simultaneously adjusting all significant variables including child age, sex and place of residence in the regression analysis

C, children less than 2500g are small

D, an aggregated index based on household assets
